# Promoter- and cell-specific epigenetic regulation of CD44, Cyclin D2, GLIPR1 and PTEN by Methyl-CpG binding proteins and histone modifications

**DOI:** 10.1186/1471-2407-10-297

**Published:** 2010-06-17

**Authors:** Imke Müller, Frank Wischnewski, Klaus Pantel, Heidi Schwarzenbach

**Affiliations:** 1 Department of Tumour Biology, University Medical Center Hamburg-Eppendorf, Martinistrasse 52, 20246 Hamburg, Germany

## Abstract

***Background*:**

The aim of the current study was to analyze the involvement of methyl-CpG binding proteins (MBDs) and histone modifications on the regulation of CD44, Cyclin D2, GLIPR1 and PTEN in different cellular contexts such as the prostate cancer cells DU145 and LNCaP, and the breast cancer cells MCF-7. Since global chromatin changes have been shown to occur in tumours and regions of tumour-associated genes are affected by epigenetic modifications, these may constitute important regulatory mechanisms for the pathogenesis of malignant transformation.

***Methods*:**

In DU145, LNCaP and MCF-7 cells mRNA expression levels of CD44, Cyclin D2, GLIPR1 and PTEN were determined by quantitative RT-PCR at the basal status as well as after treatment with demethylating agent 5-aza-2'-deoxycytidine and/or histone deacetylase inhibitor Trichostatin A. Furthermore, genomic DNA was bisulfite-converted and sequenced. Chromatin immunoprecipitation was performed with the stimulated and unstimulated cells using antibodies for MBD1, MBD2 and MeCP2 as well as 17 different histone antibodies.

***Results*:**

Comparison of the different promoters showed that MeCP2 and MBD2a repressed promoter-specifically Cyclin D2 in all cell lines, whereas in MCF-7 cells MeCP2 repressed cell-specifically all methylated promoters. Chromatin immunoprecipitation showed that all methylated promoters associated with at least one MBD. Treatment of the cells by the demethylating agent 5-aza-2'-deoxycytidine (5-aza-CdR) caused dissociation of the MBDs from the promoters. Only MBD1v1 bound and repressed methylation-independently all promoters. Real-time amplification of DNA immunoprecipitated by 17 different antibodies showed a preferential enrichment for methylated lysine of histone H3 (H3K4me1, H3K4me2 and H3K4me3) at the particular promoters. Notably, the silent promoters were associated with unmodified histones which were acetylated following treatment by 5-aza-CdR.

***Conclusions*:**

This study is one of the first to reveal the histone code and MBD profile at the promoters of CD44, Cyclin D2, GLIPR1 and PTEN in different tumour cells and associated changes after stimulation with methylation inhibitor 5-aza-CdR.

## Background

Global chromatin changes have been shown to occur in tumours. In chromosomal regions of tumour-associated genes epigenetic modifications may constitute important regulatory mechanisms for the pathogenesis of malignant transformation [1]. Inactivation of tumour suppressor genes by promoter hypermethylation has been reported for diverse tumours and is thought to play a crucial role in carcinogenesis [2]. DNA methylation affects mainly the cytosine base in a CpG dinucleotide, which is found isolated or clustered in so called CpG islands, and may induce gene repression by inhibiting the access of transcription factors to their binding sites, and by recruiting methyl-CpG binding proteins (MBDs) to methylated DNA together with histone modifications [3].

To date, five MBDs have been identified: MBD1, MBD2, MBD3, MBD4 and MeCP2. These proteins are implicated in the transcriptional repression of methylated DNA [4,5]. With the exception of MBD4, belonging to the uracil DNA glycosylase superfamily [5], the members of the family associate with histone deacetylases (HDACs). MBD1 is alternatively spliced to produce five protein isoforms (PCM1, MBD1v1, MBD1v2, MBD1v3 and MBD1v4) which differ in the number of cysteine-rich (CXXC) domains and the carboxyl-terminal sequence. Although repression of unmethylated genes has been reported to depend on the third CXXC domain [6], recent findings indicate that the two other CXXC domains may also contribute to the repression of unmethylated promoters, however, with a weaker affinity [7]. Two isoforms of MBD2 are known: MBD2a and MBD2b. The shorter form, MBD2b, starting at the second methionine lacks the N-terminal sequence of MBD2a [8]. MBD2a may act either as an activator or a repressor of transcription [7-10].

Epigenetic modifications include not only methylation of DNA but also configurational changes in chromatin which are implicated in transcriptional regulation, as well. The N-terminal tails of histones are subject to post-translational modifications, such as acetylation, phosphorylation, ubiquitination and methylation. Histone acetylation may be a predominant mark in active chromatin regions, and acetyl groups are removed by HDACs. Methylation of the lysine residue 4 of histone H3 (H3K4) is highly conserved and associated with transcriptionally active genes. Methylation of the lysine residue 9 of histone H3 (H3K9) recruits the heterochromatin protein HP-1, which condenses the chromatin into an inactive conformation. Both, DNA methylation and histone modifications may be linked by MBDs. Nearly all members of the family can interact with histone methyltransferases and deacetylases [11].

Tumour invasion is accompanied by migration of malignant cells into the surrounding connective tissue [12]. Alterations in cell-cell and cell-matrix interactions are involved in this process. CD44 is a glycoprotein and main receptor for hyaluronic acid, collagen, fibronectin and osteopontin, and regulates the cytoskeleton by transduction of signals from the extracellular matrix. Moreover, CD44 is involved in leukocyte binding to vascular endothelium at sites of inflammation [13]. Numerous isoforms of CD44 exist, and some of them are overexpressed on breast tumour cells which seems to be correlated with the metastatic potential [14]. Furthermore, the phenotype of breast tumour cells showed that CD44 may distinguish tumour-initiating from non-tumourigenic cells [15]. Recent experimental and clinical investigations showed that CD44 together with heparanase and hyaluronan regulates tumour cell proliferation, migration, invasion and angiogenesis and associates with breast cancer patient survival [16]. In respect to the down-regulation of CD44 during progression and metastasis of prostate cancer, CD44 is a metastasis suppressor for this tumour type [17]. Aberrant promoter hypermethylation has been described for CD44 gene silencing [18].

The D-type cyclins D1, D2 and D3 and their associated cyclin-dependent kinases are critical components for cell proliferation. They are expressed during the cell cycle at G0/G1-S-transition. Cyclin D2, implicated in cell differentiation and malignant transformation, is inactivated by promoter hypermethylation in several human cancers. High DNA methylation levels of Cyclin D2 cause deregulation of the G1/S checkpoint, and correlate with clinicopathologic features of tumour aggressiveness in breast and prostate cancer [19,20].

GLIPR1 (glioma pathogenesis-related protein 1) is a novel p53-target gene [21] cloned from human glioblastoma cell lines and its expression in astrocytic tumours correlated with tumour grade [22]. In contrast to its oncogenic effect in glioma, where GLIPR1 regulates proliferation, migration and survival of glioma cells, it acts as a tumour suppressor in prostate cancer. Down-regulation in this context appears to be caused by epigenetic rather than genetic changes [23].

PTEN (phosphatase and tensin homologue) is a well-known tumour suppressor that inhibits cell proliferation and migration by antagonizing the phosphatidylinositol 3-kinase (PI3K) signaling pathway [24]. In many primary and metastatic human tumours PTEN is inactivated by mutations, deletions or promoter hypermethylation [25,26].

In the present study, the promoters of the four above-described tumour-associated genes (CD44, Cyclin D2, GLIPR1 and PTEN) were examined for methylation-dependent gene regulation, the participation of MBDs in gene silencing and the histone modifications associated with the respective promoter areas. Comparison of the settings at the promoters among each other and between different cellular contexts show a MBD-mediated promoter- and cell-specific repression of the four genes. Our data provide new insights on the histone signature at the promoters of these genes, and deliver valuable information on their epigenetic regulatory mechanism.

## Methods

### Cell Culture

All cell lines were obtained from ATCC. Prostate carcinoma cells DU145 and LNCaP, and breast adenocarcinoma cells MCF-7 were maintained in Dulbecco's modified Eagle's medium (DMEM, Invitrogen, Karlsruhe, Germany), supplemented with 10% fetal calf serum (FCS) and 2 mM L-glutamine (Invitrogen) and cultured under standard conditions (37°C, 5% CO_2_, humidified atmosphere). Cell viability was determined by trypan blue staining. Each cell line was stimulated by 5-aza-2'-deoxycytidine (5-aza-CdR, f.c. 1 μM, Sigma-Aldrich, Taufkirchen, Germany) for 72 h. 5-aza-CdR-treated cells or a mock control were stimulated by Trichostatin A (TSA, f.c. 500 nM, Sigma-Aldrich) for the last 24 h of the 72 h incubation.

### mRNA expression analysis

To determine the mRNA expression of CD44, Cyclin D2, GLIPR1 and PTEN, total RNA was extracted from DU145, LNCaP and MCF-7 cells using the RNeasy^® ^Mini Kit (Qiagen, Hilden, Germany) according to the manufacturer's description. Synthesis of cDNA was carried out using the SuperScript First strand System with random hexamer primers (Invitrogen). PCR amplification of cDNA was performed with primers specific for CD44: 5' (forward) GTGATCAACAGTGGCAATGGA and 3' (reverse) TCACCAAATGCACCATTTCCT (PCR product 94 bp), Cyclin D2: 5' TGGGGAAGTTGAAGTGGAAC and 3' ATCATCGACGGTGGGTACAT (175 bp), GLIPR1: 5' TGCCAGTTTTCACATAATACAC and 3' GGATTTCGTCATACCAGTTT (142 bp), PTEN: 5' TTGAAGACCATAACCCACCACAG and 3' GGCAGACCACAAACTGAGGATTG (387 bp), β-Actin: 5' GGCGGCACCAGCATGTACCCT and 3' AGGGGCCGGACTGGTCATACT (202 bp). The reaction was performed in a final volume of 20 μl containing PCR Buffer (Qiagen), 200 μM of each dNTP (Roche Applied Science, Mannheim, Germany), 0.5 μM of each primer and 2.5 units of Taq polymerase (Qiagen). After a PCR run for 30 cycles on a Peltier Thermal Cycler (PTC-200, Biozym, Oldendorf, Germany), the PCR products were electrophoretically separated on a 1% agarose gel.

### Bisulfit Genomic Sequencing

Genomic DNA was isolated from the cultured cells using the QIAamp DNA Mini Kit (Qiagen) according to the manufacturer's description. Approximately 0.5-1 μg genomic DNA was bisulfite-converted and purified according to the recommended protocol of the EpiTect Bisulfite Kit (Qiagen). One μl of converted DNA was amplified and sequenced by the following primers: CD44 5' (forward) TGTGAAATTTAGAGATTTTGTTTTAG and 3' (reverse) AAATTTTAAAAAATAACAACCCTCCC, Cyclin D2 5' GGGTTAGTTGTTGTTTTTTTTAATAA and 3' AAAAAAATTTTTCTATTTTTATTTTT, GLIPR1 5' TTATTATGTGTTGATATGATTTTAAAAAG, and 3' AACCCACAACTTTACAAACCTAACC, PTEN 5' GTTTTTTTTGAAAGGGAAGGTG and 3' CAAACCCCCTCCCTAAAACTA. Sequencing amplification was run using BigDye reagent and buffer (Amersham Biosciences, Freiburg, Germany). After ethanol precipitation of the PCR products the pellets were resuspended by HiDi formamide (Applied Biosystems) and sequenced on a Genetic Analyzer 3130 (Applied Biosystems).

### MBD protein expression analysis

Protein levels of MBD1, MBD2 and MeCP2 in basal and stimulated DU145, LNCaP and MCF-7 cells as well as MBD1 knock out (MBD1^-/-^) mouse embryonic fibroblasts (MEF) were investigated by Western blot analysis as recently described using nuclear extracts and antibodies recognizing these epigenetic factors [7].

To test the specificity of the antibodies against modified histones, acidic protein extraction was performed according to a special protocol. Briefly, cells were resuspended in TEB buffer (PBS containing 0.5% v/v Triton X100 and 2 mM PMSF) and incubated for 10 min on ice. Acidic extraction was carried out in 0.2 N HCl in a rotation shaker at 4°C over night. The histone protein content in the supernatant was measured according to Bradford.

Twenty-five μg of protein extracts were separated by a 12% SDS polyacrylamide gel and transferred onto the nitrocellulose membrane Hybond-C extra (Amersham). After blocking, the membrane was probed with a 1:500 or 1:1000 dilution of antibodies which are directed against the following human proteins: MBD1, MeCP2 (Abcam, Cambridge, UK), MBD2, monomethylated lysine 9 of histone H3 (H3K9me), dimethylated lysine 9 of histone H3 (H3K9me2), trimethylated lysine 9 of histone H3 (H3K9me3), monomethylated lysine 4 of histone H3 (H3K4me), dimethylated lysine 4 of histone H3 (H3K4me2), trimethylated lysine 4 of histone H3 (H3K4me3), monomethylated lysine 20 of histone H4 (H4K20me), dimethylated lysine 20 of histone H4 (H4K20me2), trimethylated lysine 20 of histone H4 (H4K20me3) (Millipore, Schwalbach, Germany).

Detection of the proteins was carried out using a peroxidase-conjugated secondary antibody (Sigma-Aldrich) and the chemiluminescence ECL detection Kit (Amersham).

### Construction of Plasmids

Reporter plasmids were constructed by cloning CD44 (-908/-118), Cyclin D2 (-507/-30), GLIPR1 (-521/-142) and PTEN (-737/-41) promoter fragments into the *Xho*I and *Hind*III sites of the pGL2-Luciferase reporter plasmid (Promega, Mannheim, Germany). For the assay targeting the Gal4-linked transcriptional repressor domain (TRD) of the MBDs to the Gal4 DNA-recognition motif, five Gal4 sequences were inserted into the *Mlu*I and *Xho*I sites directly upstream of the cloned promoter fragments. All clones were verified by enzymatic digestion and DNA sequencing.

The construction of the expression plasmids encoding the full length proteins MBD1 (isoforms MBD1v1 and MBD1v3), MBD2 (isoforms MBD2a and MBD2b) or MeCP2, and the expression plasmids containing sequences encoding a fusion protein consisting of the Gal4 DNA binding domain and the TRD of MBD1 (amino acids 383-605, MBD1-TRD), MBD2 (45-262, MBD2-TRD) or MeCP2 (196-486, MeCP2-TRD) has been previously described [7,8].

### In vitro *methylation of plasmid DNA*

Twenty μg reporter plasmid containing CD44, Cyclin D2, GLIPR1 and PTEN promoter fragments were methylated by the *Hpa*II or *Sss*I methylase (New England Biolabs, NEB) for 4 h at 37°C using the methyl donor SAM (S-Adenosyl methionine, NEB). Efficient and complete methylation of the plasmid DNA was confirmed by its resistance to digestion with the methylation-sensitive restriction enzyme *Hpa*II. A control digestion with the isoschizomer *Msp*I was performed.

### Transfection and Luciferase reporter assay

The DU145, LNCaP and MCF-7 cells as well as the MBD1^-/- ^mouse embryonic fibroblasts [7] were transiently transfected with 0.5 μg of reporter plasmids and expression plasmids using the FuGENE Reagent (Roche Applied Science). For efficiency control 0.2 μg of a vector encoding for the Renilla Luciferase (Promega, Mannheim, Germany) was co-transfected. After 48 h incubation, the transfected cells were lysed using the Dual-Luciferase Reporter Assay System (Promega). Promoter-driven luciferase activity was measured on a 20/20^n ^Luminometer (Turner Biosystems, Sunnyvale, USA) and normalized by the Renilla Luciferase activity. Each transfection experiment was carried out in triplicate wells and repeated at least twice.

### Chromatin Immunoprecipitation assay (ChIP)

DU145, LNCaP and MCF-7 cells at 80% confluence were fixed with 1% formaldehyde in minimal medium for 10 min at room temperature. The DNA/protein cross linking reaction was stopped by adding a glycine stop-fix solution. The cells were washed with ice cold PBS, scraped and pelleted by centrifugation for 10 min, 4°C, at 720 g. Cells were lysed with a hypotonic lysis buffer, and the nuclei were pelleted by centrifugation for 10 min, 4°C, at 2400 g. The nuclei pellet was sheared in 1 ml shearing buffer by sonication at 25% power for 4 min on ice (Sonicator UP50H, Dr. Hielscher GmbH, Teltow, Germany) to chromatin fragment lengths of 200 to 1000 bp. The stop-fix solution, hypotonic lysis buffer and shearing buffer were obtained from the ChIP-IT kit (Active Motif, Rixensart, Belgium). The chromatin extract was pre-cleared with protein G beads. 170 μl aliquots of the supernatant were immunoprecipitated using 3 μg of the antibodies specific for IgG (Active Motif), the antibodies against acetylated histones H2A (K5), H2B (K12), H3 (K9), H4 (K8) and non-modified histones H2A, H2B, H3, H4 (Cell Signaling, Danvers, USA), and the antibodies as described above, overnight at 4°C. The DNA/protein/antibody complexes were incubated with protein G beads for 2 h at 4°C. After washing the beads, the immunoprecipitated DNA was eluted from the beads by 100 μl elution buffer containing 1% SDS and 50 mM NaHCO_3 _for 15 min, and protein-DNA crosslinks were reversed with 200 mM NaCl by incubation at 65°C for 4 h. Digestion of the proteins was performed with 0.1 mM EDTA, 20 mM Tris-HCl pH 6.5 and 2 μl Proteinase K solution (Active Motif) for 2 h at 42°C. The DNA was purified by mini-columns (Active Motif).

### Quantitative real-time PCR

The immunoprecipitated DNA fragments were amplified by the following primer pairs: CD44 5' (forward) TCTCTCCAGCTCCTCTCCCAG and 3' (reverse) GACAGAGGATGACCGAACCG (147 bp), Cyclin D2 5' GCTTCAGAGCGGAGAAGAGC and 3' GCAGAGAGAGAAGGTGGAGCAG (139 bp), GLIPR1 5' TTCTGAAAGCATTTTGCGAGG and 3' TTTAATGGAGGTTGCGGTGATA (73 bp), PTEN 5' GGGTCTGAGTCGCCTGTCAC and 3' GACCAACTCTCCGGCGTTC (54 bp), RPLP0 5' TTAGTTTGCTGAGCTCGCCAG and 3' CTCTGAGCTGCTGCCACCTG (97 bp).

To quantify the mRNA expression in the cell lines the following primers were used: CD44 5' CCCAGATGGAGAAAGCTCTG and 3' GTTGTTTGCTGCACAGATGG (113 bp), Cyclin D2 5' TTCCGCAGTGCTCCTACTTC and 3' CGCACTTCTGTTCCTCACAG (105 bp), GLIPR1 5' CTGTGGCCACTACACTCAGG and 3' AGAGCGTCAAAGCCAGAAAC (95 bp), PTEN 5' CCCAGACATGACAGCCATC and 3' TCTGCAGGAAATCCCATAGC (126 bp), RPLP0 5' ACCCAGCTCTGGAGAAACTGC and 3' TGAGGTCCTCCTTGGTGAACA (72 bp). The PCR reaction contained 2 μl template, 7.5 μl SYBRGreen Mastermix (Qiagen), and 4 pmol primer sets (Sigma-Aldrich, München, Germany) in a final volume of 15 μl, and was carried out at a melting temperature of 60°C and in 45 cycles on a Realplex System (Mastercycler epgradient S, Eppendorf, Hamburg, Germany). A dilution series of 10, 2.5, 1.25, 0.3125 and 0.078 ng/μl template DNA served as internal standard for quantification. All experiments were done in triplicate and each PCR was repeated at least twice. Evaluation of the data was performed by the Realplex software.

### Statistical analysis

The statistical analyses were performed using the SPSS software package, version 13.0 (SPSS Inc. Chicago, IL). Student's t-test and Fisher's exact test were used to identify possible statistical differences in activation and repression of gene expression, binding affinities of MBDs and histone modifications between basal and stimulated cell lines. Analysis of variance (ANOVA) was performed to determine if the means of several groups are likely to be equal.

The analyses were explorative and generated hypotheses that have to be validated in further studies. Therefore, no adjustment for multiple testing like a Bonferroni correction was performed.

The diagrams are based on the mean values of measured values. The error bars represent the standard deviation (STDEV).

## Results

### mRNA expression of CD44, Cyclin D2, GLIPR1 and PTEN in DU145, LNCaP and MCF-7 cells prior to and after treatment with 5-aza-2'-deoxycytidine and/or Trichostatin A

**Figure 1:**
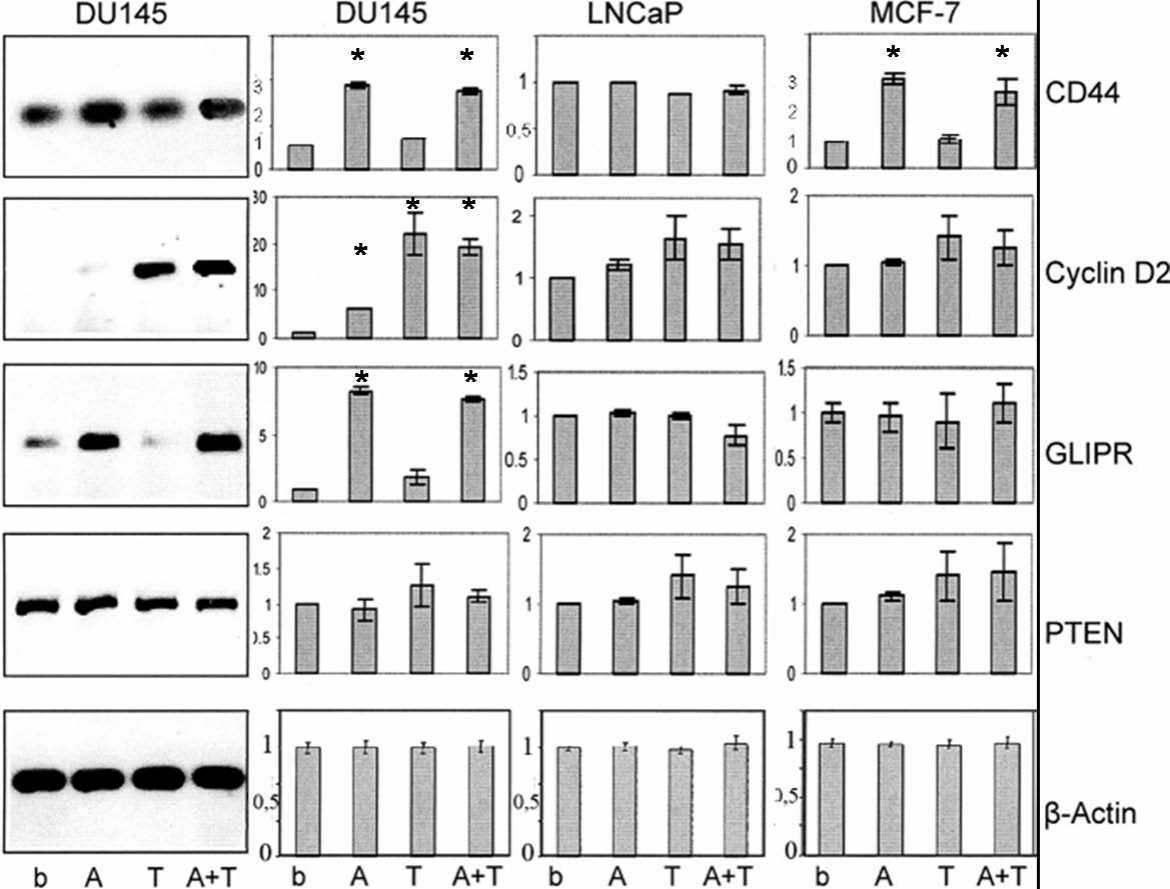
**mRNA expression of CD44, Cyclin D2, GLIPR1 and PTEN in DU145, LNCaP and MCF-7 cells**. The data assessed by RT-PCR (gel electrophoretically separated on a representative agarose gel, left panel) and quantitative real-time PCR (bar charts, right panels) show the relative levels and changes in the mRNA expression of the tumour-associated genes in unstimulated basal (b), 5-aza-CdR- (A), TSA- (T) and 5-aza-CdR+TSA- (A+T) stimulated DU145, LNCaP and MCF-7 cells. The housekeeping gene β-Actin was selected as internal control. * Statistical significance of p < 0.05 according to the Fisher's exact test in respect of changes in stimulated samples compared to the basal status.

Basal mRNA expression of CD44, Cyclin D2, GLIPR1 and PTEN was determined by reverse transcription-PCR and quantitative real-time PCR using gene-specific primers. To measure the influence of DNA methylation and histone deacetylation the cells were also incubated with the demethylating agent 5-aza-2'-deoxycytidine (5-aza-CdR) and/or the histone deacetylase inhibitor Trichostatin A (TSA). In Figure 1, the basal mRNA levels in the cell lines are depicted in comparison to the cells treated with 5-aza-CdR and/or TSA.

CD44 was constitutively expressed in LNCaP cells, and 5-aza-CdR and TSA had no further influence on the expression level. In contrast, in DU145 and MCF-7 cells the transcription of CD44 could be significantly up-regulated by 5-aza-CdR, whereas TSA had no activating effect. These findings suggest that in basal LNCaP cells the CD44 promoter is unmethylated, whereas in basal DU145 and MCF-7 cells CD44 promoter activity may be repressed by DNA methylation.

In LNCaP and MCF-7 cells Cyclin D2 was constitutively expressed. Amazingly, TSA had a significantly stronger effect than 5-aza-CdR on the expression of Cyclin D2 in DU145 cells indicating potential inactive histone modifications at the promoter.

GLIPR1 was also constitutively expressed in LNCaP and MCF-7 cells, while 5-aza-CdR stimulation was significantly sufficient for a high re-expression of GLIPR1 in DU145 cells.

The basal transcriptional activity of PTEN could not be further up-regulated by the agents in all cell lines tested.

### Methylation status of the CD44, Cyclin D2, GLIPR1 and PTEN promoters

To define the methylation status of the four promoters in the three cell lines bisulfite genomic sequencing was performed. The promoters of the highly expressed genes, such as PTEN in all cell lines, CD44 in LNCaP and Cyclin D2 in LNCaP and MCF-7, were unmethylated. Sixty percent of the CpG sites of the GLIPR1 promoter were methylated in basal DU145 cells, and treatment of these cells by 5-aza-CdR led to a decrease in methylation and activation of gene expression. The Cyclin D2 promoter was hardly methylated (6%) in basal DU145 cells, which parallels with the resistance to 5-aza-CdR.

### Repression of CD44, Cyclin D2, GLIPR1 and PTEN by MBD1, MBD2 and MeCP2 in DU145, LNCaP and MCF-7 cells

**Figure 2:**
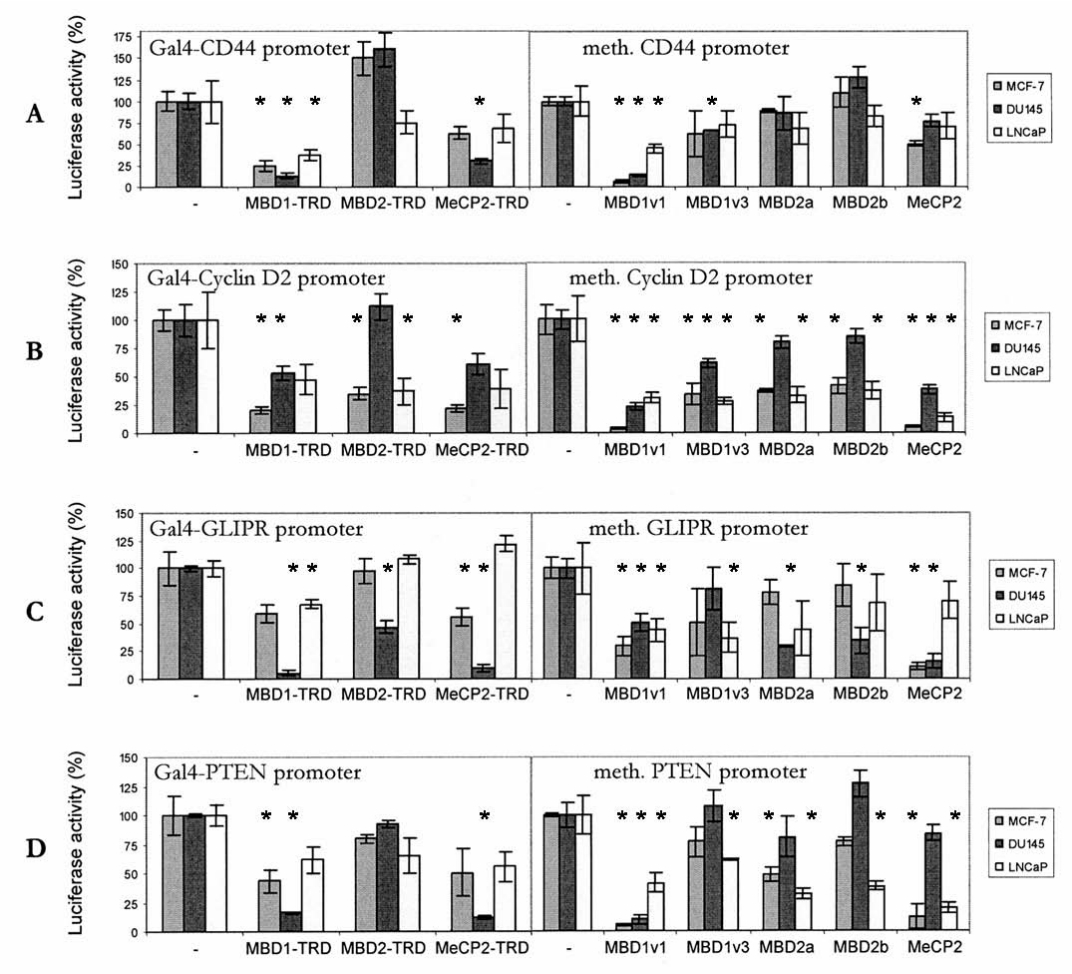
**Luciferase activities of the co-transfected reporter plasmids containing the promoters of CD44, Cyclin D2, GLIPR1 or PTEN in MCF-7, DU145 and LNCaP cells**. MCF-7, DU145 and LNCaP cells were transiently transfected with pGL2-Luciferase reporter plasmids containing promoter fragments of CD44 (A), Cyclin D2 (B), GLIPR1 (C) or PTEN (D) immediately downstream of five Gal4 binding motifs (left diagrams), or with methylated reporter plasmids containing the same promoters without the Gal4 motifs (right diagrams). The co-transfected expression plasmids encoded either for Gal4-fused TRDs of MBD1, MBD2, or MeCP2 (left diagrams) or for full length proteins of MBD1v1, MBD1v3, MBD2a, MBD2b or MeCP2 proteins (right diagrams). The activities derived from the reference Renilla Luciferase were used for normalization of the data. The relative luciferase activities of the reporter constructs co-transfected with the empty expression plasmids (-) were arbitrarily set to 100%. * Statistical significance of p < 0.05 according to the Fisher's exact test in respect of repression of promoters by MBDs compared to the basal level activity (-).

To functionally monitor the effect of the different MBDs on CD44, Cyclin D2, GLIPR1 and PTEN regulation, we performed transient co-transfection experiments in DU145, LNCaP and MCF-7 cells (Figure 2). Two different assays were used: First, the luciferase activity was measured after targeting the Gal4-linked transcriptional repressor domain of MBD1, MBD2 and MeCP2 (MBD1-TRD, MBD2-TRD and MeCP2-TRD) to the Gal4 DNA-recognition motif upstream the CD44, Cyclin D2, GLIPR1 and PTEN promoter fragments (Figure 2, left diagrams). Secondly, to more precisely define the role of the MBDs, we co-transfected full length MBD1v1, MBD1v3, MBD2a, MBD2b and MeCP2 together with methylated and unmethylated reporter constructs, and assessed their influence on transcription in response to methylation by *Sss*I (data not shown) and *Hpa*II (Figure 2, right diagrams).

Immunoblot analysis using antibodies specific for MBD1, MBD2 and MeCP2 documented efficient protein expression in the transfected cells (data not shown).

Evaluation of promoter-driven luciferase activities shows that DNA methylation of the reporter plasmids by *Sss*I caused a stronger decrease of the CD44, Cyclin D2, GLIPR1 and PTEN promoter activity than that by *Hpa*II due to the higher number of *Sss*I sites than *Hpa*II sites in the promoters (data not shown).

The Gal4 domain-mediated binding of MBD1-TRD to the Gal4 motif upstream of the promoters led to a significant repression of luciferase activity with all promoters (CD44, Cyclin D2, GLIPR1 and PTEN) in nearly all transfected cells (Figure 2, left diagrams). These findings indicate that MBD1 may have a general repressive effect on these tumour-associated genes. However, MBD2-TRD linked by the fusion part of the Gal4 DNA binding domain to the Gal4 sequence had a variable effect on the particular promoters and in the different tumour cells. While MBD2-TRD did not suppress the promoter of CD44 in all cells used, it was able to significantly repress Cyclin D2 in LNCaP and MCF-7 cells, and GLIPR1 in DU145 cells. Moreover, MBD2-TRD had only a slightly repressive effect on the basal expression of PTEN in LNCaP cells. With exception of the promoters of CD44 and GLIPR1 in LNCaP cells, MeCP2-TRD could repress to a different extent all investigated genes in all cells (Figure 2, left diagrams).

In order to cover more informative aspects of the regulation of the four tumour-associated genes, co-transfections using expression plasmids, which encode for the full length MBD proteins, and unmethylated and *Hpa*II-methylated reporter plasmids were accomplished without using the artificial link by the Gal4 system. Taken together, the results of these transient co-transfections largely support the data of the co-transfections based on the Gal4 system, with the exception of the promoters of PTEN and Cyclin D2. Here, in contrast to the Gal4-mediated binding, MBD2a and MeCP2 had a repressive effect on the methylated PTEN promoter in LNCaP and MCF-7 cells, and MBD2a suppressed the Cyclin D2 promoter in all cell lines tested (Figure 2B and 2D, right diagrams). Usually, MBD1v1 and MBD2a had a stronger influence on the promoter activity than MBD1v3 and MBD2b, respectively (Table 1). Moreover, co-transfections using the unmethylated reporter plasmids show that in contrast to the other members of the MBD family only MBD1v1 was able to repress the activity of the unmethylated promoters (data not shown and Table 1). The ability of MBD1v1 to bind unmethylated DNA depends on its third CXXC domain [6]. Although the isoform MBD1v3 has only two of these domains, it could slightly repress the unmethylated promoters (Table 1), which was also observed for other promoters [7].

**Table 1:** MBD-mediated repression of CD44, Cyclin D2, GLIPR1 and PTEN

*Repression (%)*		Gal4-linked promoter Gal4BD-TRDs			*methylated promoter* *full length proteins*					unmethylated promoter full length proteins	
		MBD1	MBD2	MeCP2	*MBD1v1*	*MBD1v3*	*MBD2a*	*MBD2b*	*MeCP2*	MBD1v1	MBD1v3
CD44	MCF-7	55	-	20	*85*	*5*	*-*	*-*	*45*	75	-
	DU145	75	-	55	*80*	*25*	*-*	*-*	*10*	75	25
	LNCaP	30	-	-	*35*	*-*	*-*	*-*	*-*	70	20

Cyclin D2	MCF-7	65	50	65	*90*	*45*	*50*	*45*	*90*	75	50
	DU145	30	-	15	*65*	*30*	*5*	*-*	*50*	20	-
	LNCaP	15	25	20	*45*	*50*	*40*	*40*	*65*	30	-

GLIPR	MCF-7	20	-	20	*50*	*10*	*-*	*-*	*80*	40	-
	DU145	85	45	85	*35*	*-*	*60*	*45*	*65*	50	-
	LNCaP	20	-	-	*20*	*25*	*-*	*-*	*-*	20	-

PTEN	MCF-7	30	-	15	*95*	*10*	*45*	*20*	*75*	75	25
	DU145	80	-	85	*75*	*-*	*-*	*-*	*-*	50	25
	LNCaP	20	10	20	*30*	*20*	*45*	*35*	*55*	65	-

-, no repression

**Figure 3:**
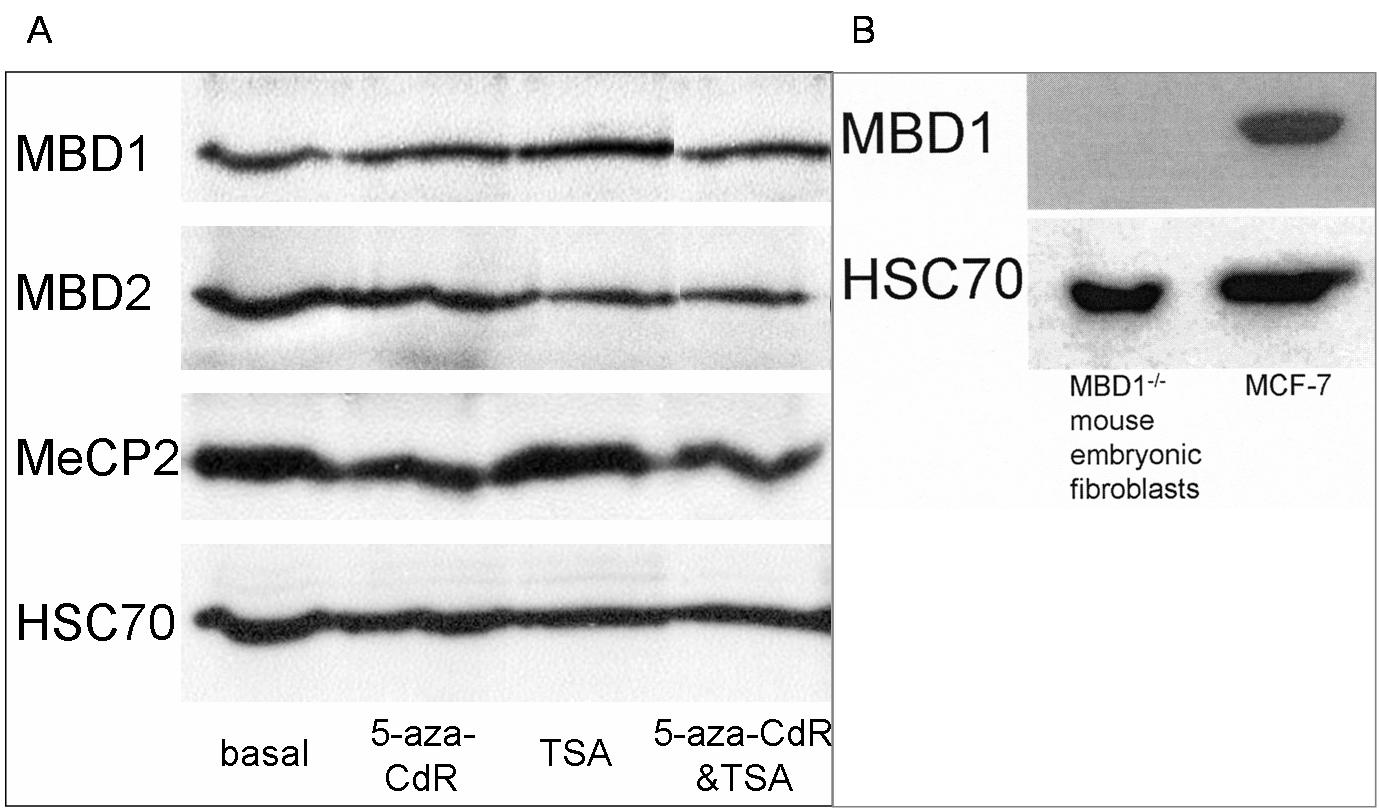
**Protein expression of MBD1, MBD2 and MeCP2 in basal and stimulated MCF-7 cells and in MBD1^-/- ^mouse embryonic fibroblasts**. The MBD protein levels in basal, 5-aza-CdR-, TSA- and 5-aza-CdR&TSA- stimulated MCF-7 cells were evaluated by Western Blot analysis using antibodies specific for MBD1 (60 kDa), MBD2 (49 kDa), MeCP2 (70 kDa) and HSC70 (70 kDa, loading control) (A). The MBD1 protein level in MBD1^-/- ^mouse embryonic fibroblasts was evaluated in comparison to basal MCF-7 cells with antibodies specific for MBD1 (60 kDa) and HSC70 (70 kDa, loading control) (B).

To exclude that the observed repressive effect of MBD1 is owing to endogenous MBD1 and to emphasize its role as a putative and general repressor, MBD1^-/- ^mouse embryonic fibroblasts, which do not express MBD1 (Figure 3B), were subsequently co-transfected with the reporter and expression plasmids. Transfected MBD1^-/- ^mouse embryonic fibroblasts showed comparable data to the other cell lines used and a similar repressive effect of the transfected full length MBD1 on the promoter-driven luciferase activity (Additional File 1).

### *In vivo *binding of MBD1, MBD2 and MeCP2 to the promoters of CD44, Cyclin D2, GLIPR1 and PTEN

**Figure 4:**
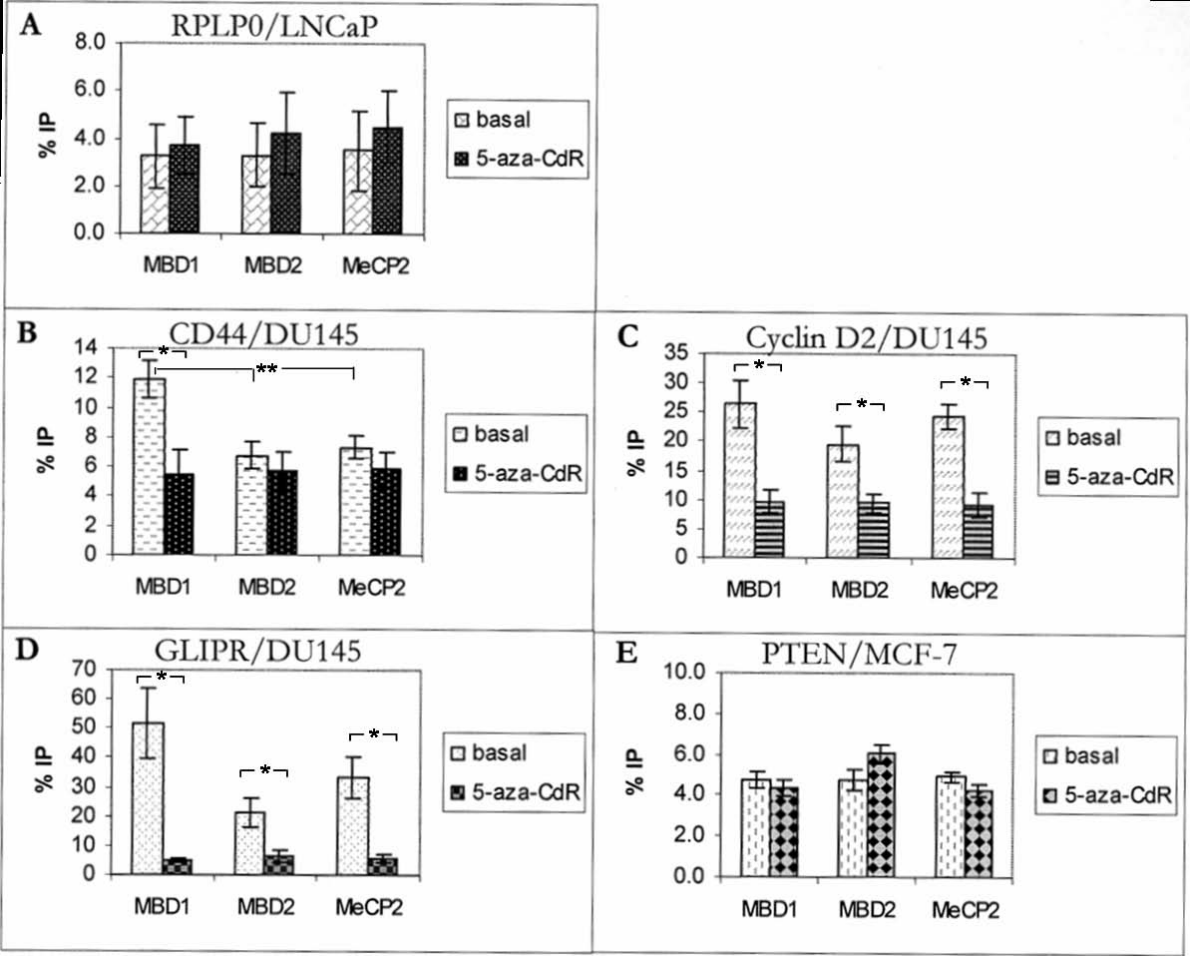
**Chromatin immunoprecipitation using antibodies specific for MBD1, MBD2 and MeCP2**. Quantitative real-time PCR analysis of the immunoprecipitated DNA (IP) derived from unstimulated (basal) and 5-aza-CdR-stimulated DU145, LNCaP and MCF-7 cells was performed using primer pairs specific for the promoter fragments of RPLP0 (A), CD44 (B), Cyclin D2 (C), GLIPR1 (D), and PTEN (E). All values obtained were normalized and referred to 100% of the input DNA. * Statistical significance of p < 0.05 according to the Fisher's exact test. ** Statistical significance of p < 0.05 according to the analysis of variance (ANOVA).

In Figure 4 five examples of the evaluation of the real-time PCR products are shown, which were representatively chosen from the data of the immunoprecipitation. To pursue the changes of the *in vivo *DNA binding of MBD1, MBD2 and MeCP2 to the promoters, the bar charts show besides the precipitated, amplified DNA derived from basal DU145, LNCaP and MCF-7 cells, also DNA from the 5-aza-CdR-stimulated cells. Due to the characteristics of a housekeeping gene, which is unmethylated and constitutively expressed, the amplified, precipitated RPLP0 (ribosomal protein, large protein 0) gene served as negative and internal control of the real-time PCR. The values of the immunoprecipitation of the RPLP0 gene were approx. 5% and used as background level (Figure 4A).

The examples of Figure 4B to 4D were chosen because of the stimulatory effect of 5-aza-CdR and TSA on the expression of these genes (Figure 1). As shown in Figure 4B, the DNA of CD44 in DU145 cells was fairly enriched by the antibody specific for MBD1 (11-13%), whereas the immunoprecipitation by MBD2 and MeCP2 was at background level. In these cells 5-aza-CdR treatment caused a significant decrease of DNA enriched by MBD1 to the background level (Figure 4B). These findings are supported by the expression analyses demonstrating that 5-aza-CdR could activate the expression of CD44 in these cells (Figure 1). Furthermore, the transfection assays sustained these data and showed that most notably MBD1v1 (80%) had a strong repressive effect on CD44, whereas MBD2a and MeCP2 had almost no influence on the methylated promoter (Figure 2A, Table 1). Similar data were obtained for CD44 in MCF-7 cells (data not shown).

As shown in Figure 4C, in DU145 cells, where Cyclin D2 was not expressed (Figure 1), an enrichment of Cyclin D2 could be observed using antibodies for MBD1 (22-30%), MBD2 (16-22%) and MeCP2 (22-27%). The stimulation of DU145 cells by 5-aza-CdR resulted in the expression of Cyclin D2 (Figure 1) and entailed a significant decrease in DNA yields of Cyclin D2 to the background level (Figure 4C). These data indicate that MBD1, MBD2 and MeCP2 are able to bind to the methylated promoter of Cyclin D2 in basal DU145 cells, and leave the promoter following administration of 5-aza-CdR to the cells. In accordance with these findings, the transfection experiments showed that MBD1v1 (65%), MBD2a (5%) and MeCP2 (50%) were able to suppress the methylated promoter of Cyclin D2 in DU145 cells (Figure 2B, Table 1).

Another example in Figure 4D demonstrates the enrichment of GLIPR1 DNA by the antibodies MBD1 (40-65%), MBD2 (15-25%) and MeCP2 (25-40%) in basal DU145 cells, and the significant decrease to the background level in 5-aza-CdR-treated cells. In DU145 cells GLIPR1 was scarcely expressed, and the stimulation of the cells by 5-aza-CdR led to a highly elevated transcriptional level (Figure 1). These findings show that the *in vivo *binding of MBD1, MBD2 and MeCP2 to the methylated promoter of GLIPR1 in basal DU145 cells is abrogated by 5-aza-CdR, and are supported by the transfection assays demonstrating the ability of MBD1v1 (35%), MBD2a (60%) and MeCP2 (65%) to repress the methylated promoter of GLIPR1 in DU145 cells (Figure 2C, Table 1).

The DNA immunoprecipitated from MCF-7 (Figure 4E), DU145 and LNCaP cells (data not shown) by the antibodies for MBD1, MBD2 and MeCP2 did not enrich the PTEN sequence and did not exceed the background range of 5%. These findings agree with those of the expression analyses where PTEN was constitutively expressed and could not be further up-regulated by 5-aza-CdR (Figure 1) suggesting that the PTEN promoter is unmethylated in these cells. The lacking occupancy of MBDs to the promoter was also observed for constitutively expressed CD44 in LNCaP cells, as well as for Cyclin D2 and GLIPR1 in LNCaP and MCF-7 cells (data not shown and Figure 1).

### Protein expression of MBD1, MBD2 and MeCP2 in DU145, LNCaP and MCF-7 cells

The distribution of the different endogenous MBDs in each cell line was scrutinized by immunoblot analyses, which show similar expression levels of MBD1, MBD2 and MeCP2 in untreated and stimulated DU145, LNCaP (data not shown) and MCF-7 cells (Figure 3A). These findings show that the different binding affinities and repressive effects of the MBDs were not caused by the different expression levels of these proteins in the various cell lines and by the stimulation of these cells. Moreover, the loss of expression of MBD1 in the MBD1^-/- ^mouse embryonic fibroblasts is demonstrated in Figure 3B.

### Histone signature at the promoters of CD44, Cyclin D2, GLIPR1 and PTEN

Promoter activity may also be regulated by numerous modifications of the histones associated with the promoter. In general, acetylation of the N-terminal histone tails is a dominant signal for active chromatin facilitating the binding of the components of the basal transcription machinery. Histone methylation can be either an active or repressive signal. Mono-, di- and trimethylation of H3K4 are involved in gene expression. In contrast, mono-, di- and trimethylation of H3K9 and H4K20 correlate with stably transcriptional repressive regions of the genome.

In order to characterize the signature of the histones, which are bound to the active and repressive promoters, ChIP assays using antibodies specific for the methylated histones H3K4, H3K9 and H4K20 as well as for unmodified and acetylated histones were accomplished. To pursue the changes in the histone signature of the promoters of CD44, Cyclin D2, GLIPR1 and PTEN in basal DU145, LNCaP and MCF-7 cells, the code was compared with that in 5-aza-CdR- or TSA-stimulated cells. DNA enriched by the antibody IgG served as negative control and background level of the respective assay.

**Figure 5:**
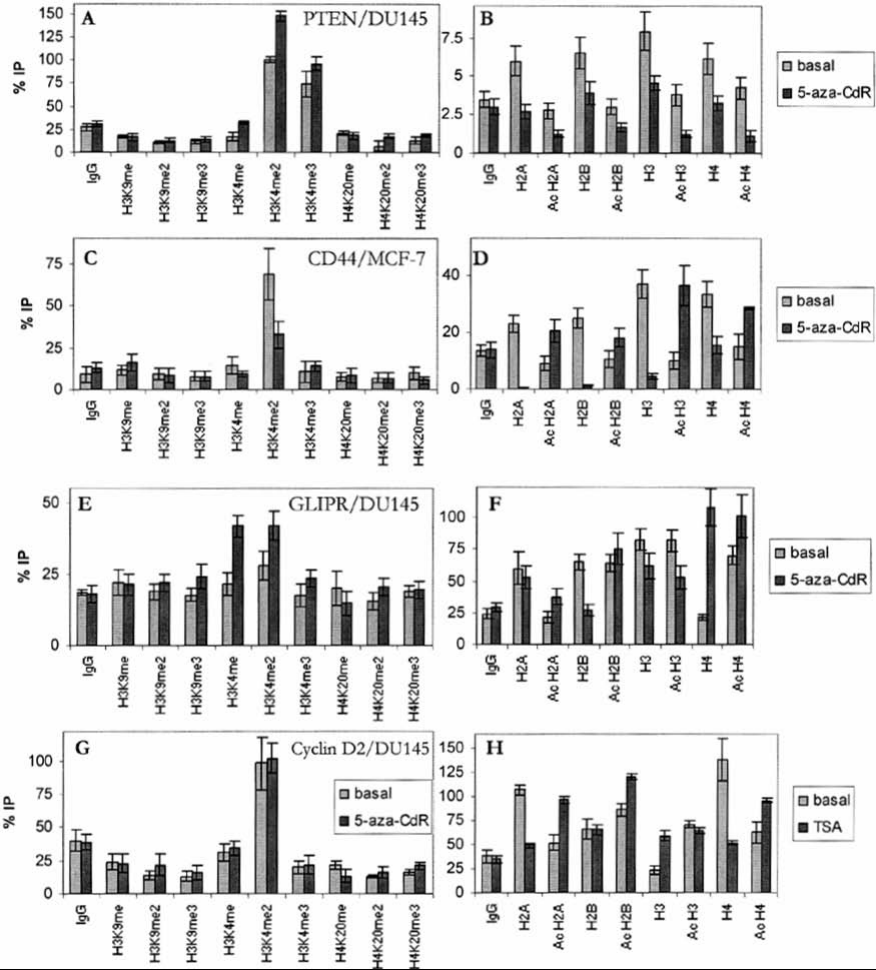
**Chromatin immunoprecipitation using antibodies specific for methylated, unmodified and acetylated histones**. Representative results of quantified DNA derived from unstimulated (basal) and 5-aza-CdR- or TSA-stimulated DU145 and MCF-7 cells immunoprecipitated by antibodies specific for methylated histones (left diagrams) as well as for unmodified and acetylated histones (right diagrams). Examples of PTEN in DU145 cells (A, B), CD44 in MCF-7 cells (C, D), GLIPR1 in DU145 cells (E, F) and Cyclin D2 in DU145 cells (G, H) are shown. All values obtained were normalized and referred to 100% of the input DNA. IgG, negative control; H3K9, Lysine 9 of histone H3; H3K4, Lysine 4 of histone H3; H4K20, Lysine 20 of histone H4; H2A, histone H2A; H2B, histone H2B; me, mono-methylated; me2, dimethylated; me3, trimethylated; Ac, acetylated.

Figures 5A and 5B show the modifications of the histones binding to the promoter of PTEN in DU145 cells. Performing the analyses of methylated histones, a specific enrichment of the DNA above background level was only detected using the antibodies for di- and tri-methylated H3K4 in the basal cells. The stimulation of the cells by 5-aza-CdR caused an increase in the immunoprecipitated dimethylated H3K4 (Figure 5A). Additionally, the analyses of the unmodified and acetylated histones showed a strengthened immunoprecipitation of the unmodified histones H2A, H2B, H3 and H4 in basal cells, which could not be observed in 5-aza-CdR-stimulated cells (Figure 5B). This histone signature in DU145 cells was similar to the code of PTEN in LNCaP and MCF-7 cells, as well as to that of CD44 in LNCaP cells and Cyclin D2 and GLIPR1 in LNCaP and MCF-7 cells (data not shown). Moreover, it might reflect the respective constitutive expression of these genes in the appropriate cell lines (Figure 1).

As shown in Figure 5C, only dimethylated H3K4 was enriched at the promoter of CD44 in basal MCF-7 cells and to a less extent in 5-aza-CdR-stimulated cells. In addition, an enrichment of the unmodified histones existed in the basal cells. However, in the 5-aza-CdR-stimulated cells this enrichment was reduced in favour of the elevated levels of the immunoprecipitated, acetylated histones H3 and H4 (Figure 5D). The acetylation of H3 and H4 might correlate with the up-regulation of the gene expression of CD44 by 5-aza-CdR in MCF-7 cells (Figure 1).

In Figure 5E a slight enrichment of dimethylated H3K4 could be observed for GLIPR1 in basal DU145 cells, whereas a considerable immunoprecipitation of mono- and dimethylated H3K4 occurred in the 5-aza-CdR-stimulated cells. It was remarkable that in 5-aza-CdR-stimulated DU145 cells more acetylated than unmodified H2B was associated with the promoter of GLIPR1. On the other hand, more unmodified than acetylated H2A was bound to the gene in the basal cells (Figure 5F). The increase in mono- and dimethylation of H3K4 and acetylation of H2B in the stimulated cells may be concordant with the high transcript level mediated by 5-aza-CdR (Figure 1). However, a high amount of acetylated H4 at the promoter of GLIPR1 was also observed in the basal cells.

As demonstrated in Figure 5G, a similar enrichment of dimethylated H3K4 could be found for Cyclin D2 in basal and treated DU145 cells. Since the promoter of Cyclin D2 could be much stronger activated by TSA than by 5-aza-CdR in DU145 cells (Figure 1) and 5-aza-CdR did not largely affect the histone signature (data not shown), an additional ChIP assay for Cyclin D2 was performed using TSA-stimulated DU145 cells. The administration of TSA to the cells led to a strong acetylation of histones H2A, H2B and H4. In contrast, unmodified H2A and H4 were enriched in basal DU145 cells (Figure 5H).

## Discussion

In the current study, the participation of MBDs in the transcriptional repression of the four selected tumour-associated genes CD44, Cyclin D2, GLIPR1 and PTEN was examined. The modifications of histones binding at the respective promoters in basal and 5-aza-CdR-stimulated prostate cancer cells DU145 and LNCaP and breast tumour cells MCF-7 were investigated, as well. Comparison of the events at the promoters of the individual genes in the different cell lines aimed to clarify whether their regulation is promoter- and/or cell-specific.

Highly constitutively expressed genes, such as PTEN in all three cell lines, Cyclin D2 and GLIPR1 in LNCaP and MCF-7 cells or CD44 in LNCaP cells are unmethylated as shown by bisulfite sequencing and could therefore not be further up-regulated by the demethylating agent 5-aza-CdR. These genes also showed no *in vivo *binding of MBDs to their promoters. However, transient transfection assays demonstrated a repressive potential of MBDs, when these promoters were methylated *in vitro*. In contrast, CD44 in DU145 and MCF-7 cells as well as Cyclin D2 and GLIPR1 in DU145 cells may be inactivated by DNA methylation. ChIP assays showed that at least one member of the MBD family bound to these promoters, and its binding affinity to the promoters correlated with its ability to repress the promoter activity in transient co-transfections. 5-aza-CdR may cause demethylation of the DNA and the release of the MBDs from the promoters. These findings show that these tumour-associated genes may be targets of therapeutic drugs, such as demethylating agents.

As described for patients with myelodysplastic syndrome (MDS) of the bone marrow, 5-aza-CdR has already been introduced as a therapeutic drug and was found to prolong survival of these patients. Besides, TSA is already used as a therapeutic drug for acute myeloid leukaemia. It can induce cell differentiation and apoptosis, has anti-proliferative effects and leads to cell-cycle arrest [27]. Combined epigenetic therapies of a demethylating agents with a histone deacetylase inhibitor indicate that these agents have significant activity in patients with MDS/acute myelogenous leukaemia [28]. Due to the findings of our study, these treatments should also be considered for patients with solid tumours.

As shown in ChIP and transient co-transfection assays, MBD1v1 bound and repressed the methylated promoters in all cell lines used. In transfection assays MBD1v1 showed additionally a repressive effect on unmethylated promoters. Based on the repressive effect of exogenous MBD1 in MBD1^-/- ^mouse embryonic fibroblasts, MBD1v1 may act as a general, epigenetic factor for these methylated promoters. The ability of MBD1v1 to bind also demethylated DNA is owing to its third CXXC domain [6]. MBD1v3 does not possess this domain, but had a weak repressive effect on the unmethylated promoters [7]. Furthermore, the ChIP assays revealed the *in vivo *binding of MBD1, MBD2 and MeCP2 to the promoter of Cyclin D2 and GLIPR1 in DU145 cells which exhibited a very low level of the respective transcripts. In contrast, CD44 was only bound by MBD1 in DU145 and MCF-7 cells in which the transcription was slightly higher than the basal expression of Cyclin D2 and GLIPR1 in DU145 cells suggesting that the promoter of CD44 might be partly demethylated. In transient co-transfection assays MeCP2 and MBD2a had a minor and rather heterogeneous influence on the methylated promoters than MBD1v1 and could inhibit gene expression in 75% and 50% of the cases, respectively. The repressive effect of MeCP2 and MBD2a on the promoter of Cyclin D2 was promoter-specific in the three cell lines, albeit the influence of MBD2a was weak in DU145 cells. In MCF-7 cells MeCP2 repressed cell-specifically the methylated promoters. Moreover, the promoters of Cyclin D2 and PTEN could be suppressed by all MBDs and their isoforms studied in LNCaP and MCF-7 cells. Our findings show that at least one member of the MBD family was always involved in repression of the methylated promoters in each cell line. These findings suggest the potential impact of therapeutical intervention on cancer patients by means of increasing expression and tumour-suppressive function of genes, which are epigenetically silenced by MBD protein occupancy.

As far as we know, only two publications have reported on such an epigenetic comparison of MBDs regarding different genes in different cell lines. In a large-scale study Lopez-Serra et al. described the binding affinity of MBDs to 22 tumour suppressor genes in 10 cell lines, among others in MCF-7 cells, in which, however, none of the four presented genes was considered [29]. These authors also referred to the binding affinity of MBD1 to unmethylated promoters. Furthermore, they showed that MBD2 and MeCP2, but not MBD1, are promoter-specific factors of the 22 genes and MBD2, but not MBD1 or MeCP2, is a cell-specific factor [29]. The promoter-specificity of MeCP2 and MBD2a in these ChIP analyses is consistent to our data based on transient co-transfection assays. The second study of Ballestar et al. investigated the binding affinity of MBDs to the promoters of 6 tumour suppressor genes in two different cell lines and normal lymphocytes using the ChIP assay. Contrary to the study of Lopez-Serra et al. and our findings this laboratory could not observe any binding of MBD1 to unmethylated as well as methylated promoters. However, they could show a gene-specific binding pattern of MBDs at the methylated promoters. Whereas MeCP2 bound to all methylated promoters, MBD2 had only binding affinity to one of the promoters studied [30].

To investigate the signature of histones binding to the promoters of CD44, Cyclin D2, GLIPR1 and PTEN in basal and 5-aza-CdR-stimulated DU145, LNCaP and MCF-7 cells, 17 antibodies specific for mono-, di- and trimethylated, acetylated and unmodified histones were applied. In agreement to the association of mono-, di- and trimethylated histone H3K4 with active genes [31] the analysis of the methylation status of the histones show mainly associations with these modifications. All three methylation grades of H3K4 were reported to be localized in the region of the transcriptional start site of known genes. An increased binding of H3K4me3 could be observed at highly expressed genes. However, H3K4me1 and H3K4me2 associated at intermediately active promoters and were found rather downstream of the transcriptional start site [31,32]. The findings of this study showed that high levels of H3K4me2 and H3K4me3 or H3K4me2 alone could be confined to highly expressed genes. The intermediately active promoters were bound by H3K4me2 and in one case by H3K4me1 and H3K4me2.

Furthermore, generally no mono-, di- and trimethylated histones H3K9 and H4K20 at the promoters were detected. This may be explained by their preferred occurrence in heterochromatin and implication in gene silencing. It was reported that H3K9me3 and H4K20me3 associated with constitutive heterochromatin and stable gene repression, and H3K9me2 and H4K20me1 associated with facultative heterochromatin and temporarily inactive genes [31,32]. Moreover, Barski et al. confirmed the presence of H3K9me2 and H3K9me3 at inactive genes while they also perceived H3K9me1 at active genes [31,32]. These data support our analysis showing the exclusive binding of H3K9me1 to the active promoter of Cyclin D2 in basal and 5-aza-CdR-stimulated MCF-7 cells. However, a recent publication showed that H3K9me3 may also be enriched in numerous active promoters [33]. To sum up, the genes of CD44, Cyclin D2, GLIPR1 and PTEN are obviously located neither in constitutive nor facultative heterochromatin.

In addition, the ChIP assays presented in this work showed that, in general, unmodified histones at repressive as well as active promoters were acetylated following stimulation of the cells by 5-aza-CdR. The activation of the repressive promoters mediated by this demethylating agent sustains the fact that unmodified and acetylated histones bind preferentially within areas of repressive and active genes, respectively. Moreover, the recruitment of HDACs by MBDs to methylated DNA leads to histone deacetylation, whereas DNA demethylation promotes the release of MBDs together with HDACs and consequently histone acetylation. A high level of acetylation after 5-aza-CdR-mediated stimulation was observed for histone H3 at the promoter of CD44 in MCF-7 cells. Comparative analyses of the methylation status and chromatin structure of the p14(ARF)/p16(INK4A) promoters showed generally the presence of higher levels of acetylated H3 at unmethylated than methylated CpG dinucleotides in a series of normal and cancer cells [34]. As a result, acetylated H3 is particularly associated with transcription and involved in histone deposition and chromatin assembly [35]. In contrast, it was reported that TSA treatment induced significant acetylation of H3 at the multidrug resistance gene 1 (MDR1) but did not activate transcription of this gene [36]. Furthermore, in 5-aza-CdR-treated cells an increase of the amount of acetylated H3 at the cytomegalovirus promoter was observed. However, hypoacetylation played only a moderate role in the inactivation of this promoter [37]. This observation is similar to the present results showing that unmodified histones, which are frequently detected at inactive genes, were associated with the constitutively expressed PTEN gene. The lacking acetylation of the histones might be compensated by the high di- and trimethylation of H3K4. Although 5-aza-CdR had no effect on the constitutive expression of this gene, it affected indirectly the acetylation of the surrounding histones. In one case of Cyclin D2 in DU145 cells TSA had a stronger effect on gene expression than 5-aza-CdR. The stimulation of the cells by TSA confirmed its function as a histone deacetylase inhibitor and caused a high degree of acetylation of the unmodified histones.

However, TSA treatment has not only been shown to open the chromosomal structure increasing the accessibility of transcription factor complexes to their binding sites on the promoter and to up-regulate gene transcription. TSA, as well as 5-aza-CdR, is also involved in the post-transcriptional regulation by changing mRNA stability. TSA can reduce the half life of mRNAs and acetylate cytoplasmic proteins [38,39]. 5-aza-CdR and TSA could affect expression levels by indirect modulation of downstream gene regulatory mechanisms and alter the subcellular distribution of proteins that mediate post-transcriptional regulation. They caused the interaction between an RNA binding protein and the estrogen receptor mRNA leading to increased mRNA stability [38]. Moreover, the modulation of mRNA stability of claudin-1, a tight junction protein, by its 3'-UTR has been revealed as the major mechanism underlying HDAC-dependent claudin-1 expression [39]. To sum up, the reports show that TSA may influence the mRNA stability of tumour-associated genes in different manners, it is able to destabilize the claudin-1 mRNA [39] while in case of the cell-cycle control gene p21^WAF1 ^it stabilized the mRNA [40]. Since we only considered the impact of 5-aza-CdR and TSA on the promoter settings, we do not know the contribution of the RNA stability in the regulation of our gene set. This requires further investigation.

## Conclusions

The presented combined investigations on biologically relevant tumour-associated genes in different tumour cell types show diverse and characteristic profiles of MBD patterns and histone signatures at the promoters. These results contribute to a more comprehensive understanding of the epigenetic interplay in tumourigenesis and the role of MBDs and histone modifications in the regulation of tumour-associated genes, and may constitute valuable information in therapeutical approaches for re-expression of tumour suppressor genes as part of individual cancer treatments.

## Abbreviations

MBD: Methyl-CpG binding protein; 5-aza-CdR: 5-aza-2'-deoxycytidine; TSA: Trichostatin A

## Competing interests

The authors declare that they have no competing interests.

## Authors' contributions

IM has performed most of the experimental work and assisted writing the manuscript. FW has helped with the experimental setup and was involved in discussion of the results. KP was involved in the discussion and perused the manuscript. HS designed the whole project, coordinated the experiments and composed the manuscript. All authors have read and approved the final manuscript.

## Pre-publication history

The pre-publication history for this paper can be accessed here:

http://www.biomedcentral.com/1471-2407/10/297/prepub

## Supplementary Material

Additional File 1 **Luciferase activities of co-transfected reporter plasmids containing the methylated promoters of CD44, Cyclin D2, GLIPR and PTEN in MBD1^-/- ^mouse embryonic fibroblasts**. In nearly all cases the co-transfected construct of MBD1v1 suppresses strongly the promoter activity of the respective methylated reporter plasmid. MBD1v3 has almost no repressive effect in these cells. MBD2a, MBD2b and MeCP2 have heterogeneous effects on the different promoters. Click here for file
