# Supplementary figures and images for: Promoter- and cell-specific epigenetic regulation of CD44, Cyclin D2, GLIPR1 and PTEN by Methyl-CpG binding proteins and histone modifications
Source: BMC Cancer. 2010 Jun 17;10:297. doi: 10.1186/1471-2407-10-297 (PMC2912262; doi:10.1186/1471-2407-10-297)

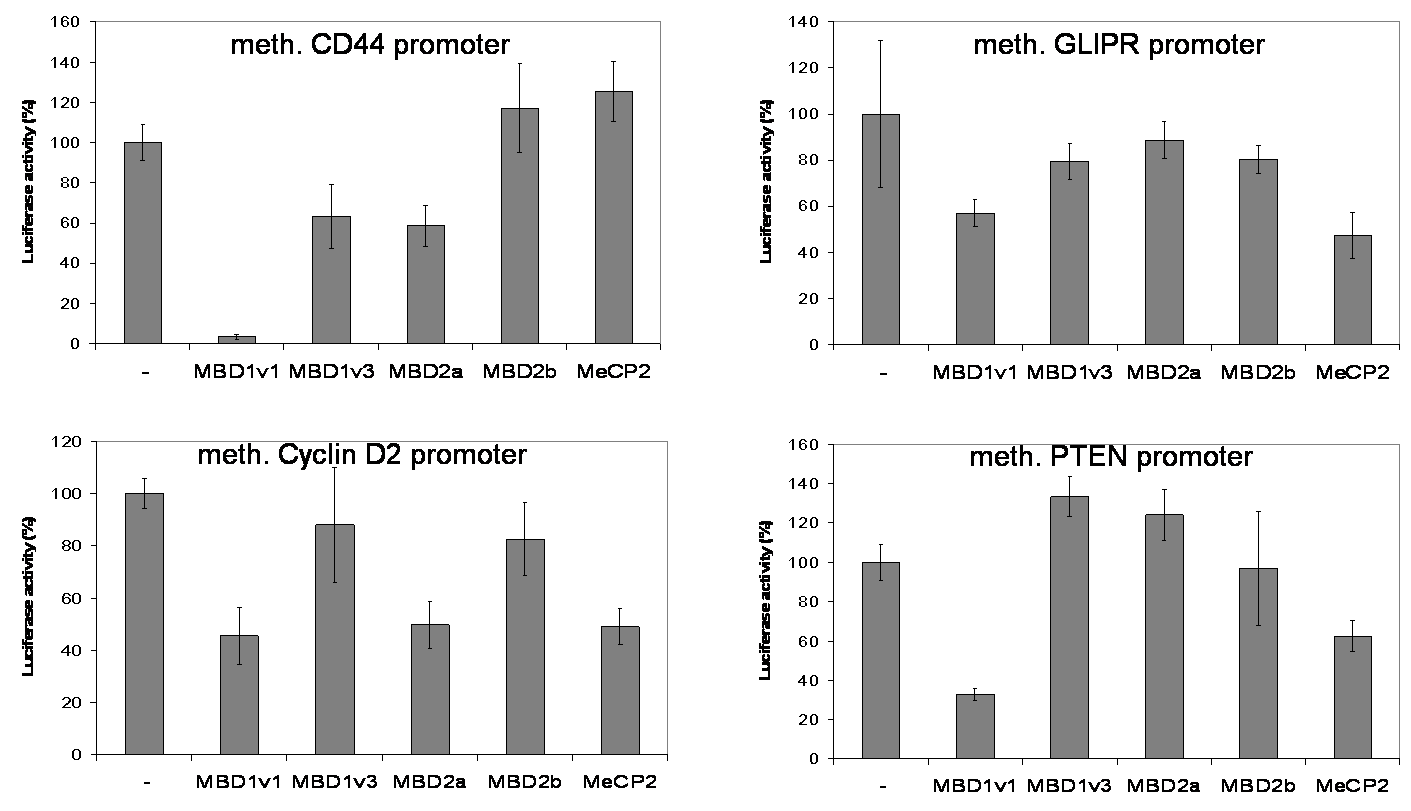

Supplement: Additional File 1 — Luciferase activities of co-transfected reporter plasmids containing the methylated promoters of CD44, Cyclin D2, GLIPR and PTEN in MBD1-/- mouse embryonic fibroblasts. In nearly all cases the co-transfected construct of MBD1v1 suppresses strongly the promoter activity of the respective methylated reporter plasmid. MBD1v3 has almost no repressive effect in these cells. MBD2a, MBD2b and MeCP2 have heterogeneous effects on the different promoters. [file 1471-2407-10-297-S1.TIFF]
